# Expression level and clinical significance of HOX transcript antisense intergenic RNA in cervical cancer: a meta-analysis

**DOI:** 10.1038/srep38047

**Published:** 2016-11-29

**Authors:** Shasha Liu, Min Zhang, Pengpeng Qu

**Affiliations:** 1 Tianjin Central Hospital of Gynecology Obstetrics, No. 156 Nankai Sanma Road Nankai District, Tianjin 300100, PR China; 2 Tianjin Medical University, 22 Qixiangtai Road, Tianjin 300070, PR China

## Abstract

The long noncoding RNA HOX transcript antisense intergenic RNA (HOTAIR) is associated with the development and progression of several types of cancer; however, its role in cervical cancer is unclear. Here, we performed a meta-analysis to evaluate the expression levels and clinical significance of *HOTAIR* in cervical cancer tissue. Relevant literature published by January 2016 was identified in PubMed, EMBase, National Knowledge Infrastructure, Wanfang Database, and Chinese Biomedical Literature Database. We performed statistical analysis, calculated standard mean differences (SMDs), and determined combined odds ratios (ORs) with 95% confidence intervals. A forest map was constructed. From the literature search, six papers (reporting 535 cases) were included from 578 papers selected according to the inclusion criteria established for the meta-analysis. The analysis showed that HOTAIR expression was significantly increased in cervical cancer compared with that in the normal control group. The ORs for tumour size and lymph node metastasis in patients with cervical cancer having high HOTAIR expression were 2.20 and 7.52, respectively. The combined hazard ratio, reflecting the influence of high HOTAIR expression in patients with cervical cancer on overall survival, was 2.56. High expression of HOTAIR affected the occurrence and development of cervical cancer.

Long noncoding RNAS (lncRNAs) are a class of diverse noncoding RNAs with lengths of more than 200 nucleotides that lack an open reading frame (ORF)^1^. LncRNAs play important roles in the proliferation, apoptosis, and invasiveness of tumour cells and affect the metastatic capacity of cancers^2^. HOX transcript antisense intergenic RNA (HOTAIR) is an lncRNA located on chromosome 12q13.13. HOTAIR has a total length of 2337 nucleotides and functions in trans regulation. With the development of deep sequencing and chip technology, studies have shown that HOTAIR is expressed in breast cancer, colon cancer, and liver cancer tissues and plays a key role in promotion of cancer and inhibition of oncogenes; however, few studies have evaluated the role of HOTAIR in cervical cancer.

Cervical cancer has the highest morbidity rates of any genital tract neoplasm in women worldwide. In China, the incidence of cervical cancer has increased annually, and patients are being diagnosed with cervical cancer at younger ages^3^,^4^. However, there is no reliable index to monitor disease recurrence and metastasis, determine prognoses, and guide individual treatments in patients with cervical cancer.

Therefore, the purpose of this study was to evaluate whether HOTAIR could be applied as a novel biomarker in the diagnosis and treatment of cervical cancer. We performed a meta-analysis of reports describing HOTAIR expression levels and clinical significance in cervical cancer. Additionally, we discussed the relationships between high HOTAIR expression and the occurrence and development of cervical cancer.

## Methods

### Literature retrieval strategy

Relevant literature was identified independently by two researchers using PubMed, EMBase, National Knowledge Infrastructure (CNKI), Wanfang Database, and Chinese Biomedical Literature Database. All studies were published by January 2016, and conflicts were solved through group discussion.

Chinese search words were “HOTAIR” and “cervical cancer”, and English search words were HOTAIR, “HOX transcript antisense RNA”, cervix (or cervical) cancer/neoplasm/tumour/carcinoma. All papers were written in Chinese or English.

### Inclusion and exclusion criteria

Studies included in this analysis had to meet the following inclusion criteria: reported expression levels of HOTAIR, as determined by quantitative reverse transcription polymerase chain reaction (RT-qPCR); provided the decision criteria for HOTAIR expression levels; divided patients into high and low expression groups; and provided data regarding the clinicopathological features of the patients.

Studies were excluded from the analysis if they met any of the following exclusion criteria: described repeated studies or included patients reported in a previous study; provided too little information; did not provide sufficient descriptions of the data; and lacked relevant data or used nonhuman samples. Reviews, letters, unpublished data, and commentaries, as well as reports presented in languages other than English or Chinese were also excluded from the analysis.

Study quality was assessed by two researchers by reading the title, abstract, and the full text of each report and referring to the inclusion and exclusion criteria.

### Data extraction

Two researchers extracted data independently using predesigned, standardised form. Extracted information included title, authors’ names, publication date, literature source, methodological information (i.e., study population, HOTAIR detection method, intercept values or methods, hazard ratios [HRs], total survival numbers, and 95% confidence intervals [CIs]), and clinical information (i.e., clinical stage, tumour size, and lymph node metastasis). Conflicts were solved through group discussion.

### Statistical methods

Homogeneity tests were performed with a significance level of α = 0.1. Homogenous data were analysed using a fixed effects model, whereas heterogeneous data were analysed using a random effects model. The combined standard mean differences (SMDs), effect scale ratios (odds ratios [ORs]), combined effects (HRs), and 95% confidence intervals (CIs) were calculated, with a significance level of α = 0.05. Statistical analysis was carried out using RevMan5.3 and Stata12.0 software packages provided by the Cochrane Collaboration Network.

## Results

### Literature search results

**Figure 1:**
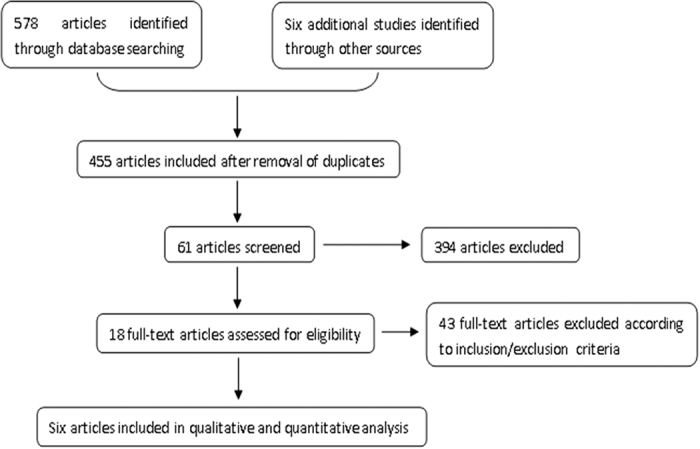
Flow diagram of the studies identified, included and excluded.

According to our search strategy, a total of 584 studies were evaluated. Of these, the following studies were excluded from analysis: 129 repeated studies, 394 articles describing unrelated topics, and 43 articles lacking relevant data. In total, six papers (containing a total of 535 cases) met the inclusion criteria and were evaluated in this study. The screening process and results are shown in Fig. 1. The basic information and data from the included studies are shown in Table 1.

### Meta-analysis results

#### Expression level of HOTAIR in cervical cancer

**Figure 2:**
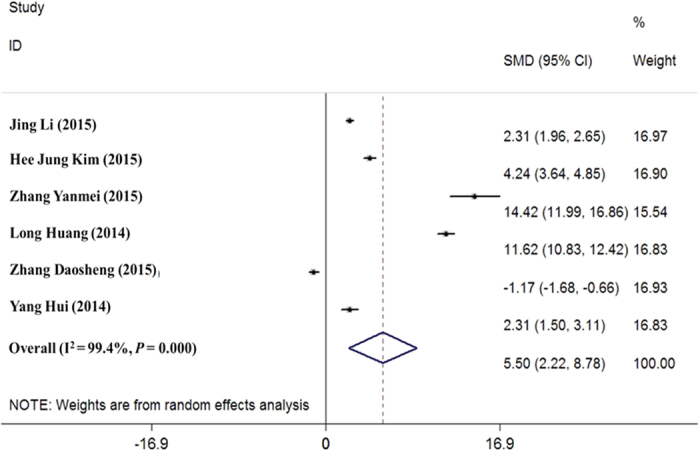
Expression of HOTAIR in cervical cancer. SMD: The horizontal scale of the solid vertical line (null line) is 0. Each horizontal line is the connection of the upper and lower bounds of the 95% confidence interval of the study. The size of the confidence interval is directly represented by the length of the line. The small squares indicate the positions of the MD or SMD value, and the size of the square is the weight of the study. If the line of the 95% confidence interval of one study crosses the dashed line, the study has no statistical significance. In contrast, if the line is on the left or right side of the null line, the study is considered statistically significant. The diamond symbol represents the combined results obtained from all of the above experiments.

A total of 535 patients with cervical cancer were included in the study. Through the meta-analysis, we found that the expression level of HOTAIR was significantly increased in patients with cervical cancer compared with that in patients in the normal control group (SMD = 5.497, 95%CI: 2.218–8.777, *P* = 0.001; random effects model, I^2^ = 99.4%), as shown in Fig. 2.

#### Effect of HOTAIR expression on lymph node metastasis in patients with cervical cancer

**Figure 3:**
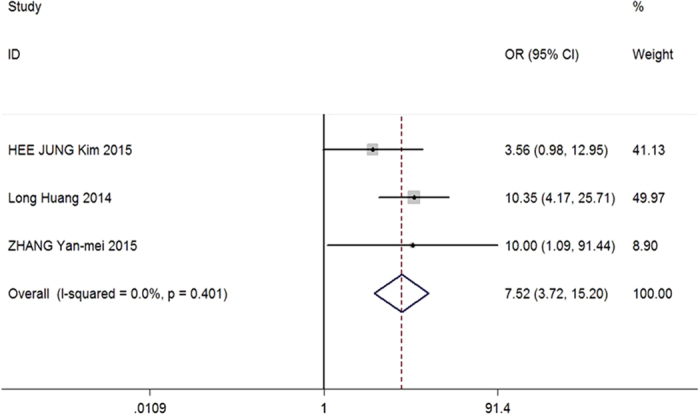
Effects of HOTAIR expression on lymph node metastasis in patients with cervical cancer. OR (forest plots):The horizontal scale of solid vertical line (null line) is 1. Each horizontal line is the connection of the upper and lower bounds of the 95% confidence interval of the study. The size of the confidence interval is directly represented by the length of the line. The small squares are the positions of the RR or OR value, and the size of the square is the weight of the study. If the line of 95% confidence interval of a study crosses the line, the study has no statistical significance. In contrast, if the line is on the left or right side of the null line, the study is considered statistically significant.

Three articles met the inclusion and exclusion criteria. For these articles, analysis of I^2^ heterogeneity showed that *P* = 0.401, I^2^ = 0%. Data were combined using a fixed effects model, and a forest map was generated. The effects of high HOTAIR expression on lymph node metastasis were then evaluated. The combined OR was 7.52 (95% CI: 3.72–15.20; *P* < 0.00001; Fig. 3). Thus, these data suggested that HOTAIR expression could be used to predict lymph node metastasis inpatients with cervical cancer.

#### Effects of HOTAIR expression on tumour size in patients with cervical cancer

**Figure 4:**
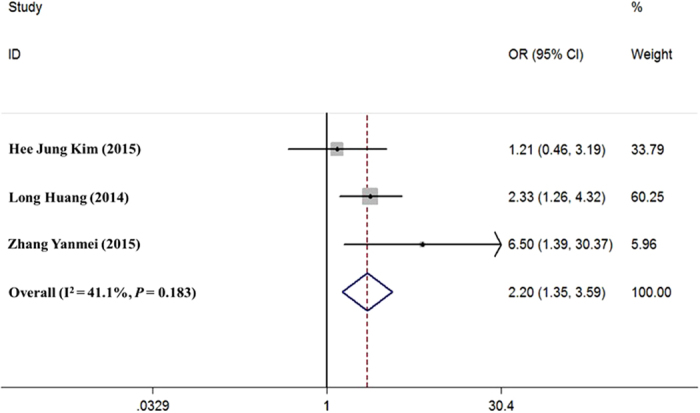
Effects of HOTAIR expression on tumour size in patients with cervical cancer. OR (forest plots): The horizontal scale of solid vertical line (null line) is 1. Each horizontal line is the connection of the upper and lower bounds of the 95% confidence interval of the study. The size of the confidence interval is directly represented by the length of the line. The small squares are the positions of the RR or OR value, and the size of the square is the weight of the study. If the line of 95% confidence interval of a study crosses the line, the study has no statistical significance. In contrast, if the line is on the left or right side of the null line, the study is considered statistically significant.

Three articles met the inclusion and exclusion criteria. For these articles, analysis of I^2^ heterogeneity showed that *P* = 0.183 (I^2^ = 41.1%). Data were combined using a fixed effects model, and a forest map was generated. The effects of high HOTAIR expression on tumour size were then evaluated. The combined OR was 2.20 (95% CI: 1.35–3.59; *P* = 0.002; Fig. 4), suggesting that the expression level of HOTAIR was associated with tumour size in patients with cervical cancer. Higher expression level of HOTAIR was associated with larger tumour.

#### Effects of HOTAIR expression on OS in patients with cervical cancer

**Figure 5:**
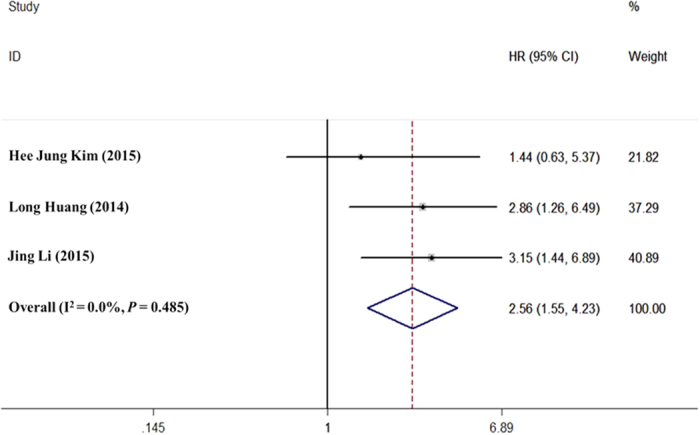
Meta-analysis of the relationship between HOTAIR expression and overall survival (OS) in patients with cervical cancer. HR (forest plots): The horizontal scale of solid vertical line (null line) is 1. Each horizontal line is the connection of the upper and lower bounds of the 95% confidence interval of the study. The size of the confidence interval is directly represented by the length of the line. The small squares are the positions of the HR value, and the size of the square is the weight of the study. If the line of 95% confidence interval of a study crosses the line, the study has no statistical significance. In contrast, if the line is on the left or right side of the null line, the study is considered statistically significant.

There was no heterogeneity among the research data; therefore, a fixed effects model was used to analyse the data. Accordingly, the effects of high HOTAIR expression on OS were evaluated. The combined HR was 2.56 (95% CI: 1.55–4.22; *P* < 0.0001; Fig. 5), suggesting that the expression level of HOTAIR was a risk factor for OS in patients with cervical cancer. That is, higher HOTAIR expression was associated with lower OS.

## Discussion

Many epidemiological and molecular biological studies have shown that genetic factors affect tumour development. In recent years, in-depth studies of lncRNAs have shown that the expression levels of lncRNAs are up- or downregulated in tumour cells. Thus, the relationship between lncRNAs and cancer has become a hot research topic^5^. Notably, lncRNAs are involved in genetic, alternative splicing, nuclear transport, and small RNA precursor functions, and disruption of lncRNA transcription and function is associated with various diseases. Additionally, lncRNAs have complex biological functions, including chromatin remodelling, X chromosome inactivation, genetic imprinting, nuclear transport, RNA splicing, and translation regulation. Thus, lncRNAs affect both physiological and pathological processes^6^.

HOTAIR was the first lncRNA found to be involved in cancer and has been shown to be upregulated in a variety of tumour tissues (including tissues from patients with liver cancer, gastric cancer, colon cancer, lung cancer, and primary and metastatic breast cancer)^7^,^8^,^9^. In this meta-analysis, we found that HOTAIR expression was significantly higher in cervical cancer tissues than in normal tissues. Notably, these results were not consistent with the findings of Zhang *et al*.^10^. These differences may be related to the sample type and detection method or to the small sample sizes of the studies.

High expression of HOTAIR may inhibit the expression of tumour metastasis-suppressor genes, thereby promoting metastasis and malignant transformation. Conversely, downregulation of HOTAIR may decrease the metastasis and invasion of cancer cells^11^. Clinical studies have shown that the expression of HOTAIR is closely related to the metastasis, recurrence, and prognosis of breast cancer, colon cancer, and liver cancer; however, few studies have examined the role of HOTAIR expression in cervical cancer^12^. In this study, the expression level of HOTAIR in cervical cancer was positively related to tumour size and lymph node metastasis. HOTAIR overexpression was a risk factor for overall survival (OS) of patients with cervical cancer. Huang and colleagues^13^,^14^,^15^ found that the expression of HOTAIR in cervical cancer is upregulated compared with that in adjacent normal tissues and that higher expression of HOTAIR was closely related to clinical stage, depth of invasion, lymph node metastasis, and tumour size. In this study, the clinical stage was not uniform and was therefore not included in the meta-analysis.

HOTAIR can play a role in mediating molecular scaffolds, promoting the localisation of polycomb repressive complex 2 and the lysine-specific demethylase 1/CoREST/REST complex to the chromatin; this can then affect chromatin histone H3K27 trimethylation and H3K4me2 demethylation, resulting in the regulation of tumour-related gene expression^2^. Thus, HOTAIR may have functions related to histone modifications of target genes. Although the exact mechanisms are still unclear, these previous studies supported the role of HOTAIR in promoting tumour invasion via alteration of chromatin^12^,^16^.

In studies of primary tumours, high expression of HOTAIR has been shown to be associated with tumour metastasis and low survival rates^17^. Notably, in our analysis, metastasis-free survival and overall survival rates were significantly lower in patients with high HOTAIR expression than in patients with low HOTAIR expression. Additionally, multivariate analysis showed that high HOTAIR expression was an independent risk factor for cervical cancer metastasis and poor prognosis. Further studies are needed to confirm these findings in greater numbers of patients.

In summary, in this meta-analysis investigating the role of HOTAIR in cervical cancer, we confirmed that abnormal expression of HOTAIR was closely related to cervical cancer development, metastasis, and invasion. HOTAIR expression also reflected the malignant development of cervical cancer, to some extent. Thus, the expression level of HOTAIR may have applications as a new biomarker in the diagnosis and treatment of cervical cancer.

## Additional Information

**How to cite this article**: Liu, S. *et al*. Expression level and clinical significance of HOX transcript antisense intergenic RNA in cervical cancer: a meta-analysis. *Sci. Rep*. **6**, 38047; doi: 10.1038/srep38047 (2016).

**Publisher's note:** Springer Nature remains neutral with regard to jurisdictional claims in published maps and institutional affiliations.

## Figures and Tables

**Table 1:** Detailed information on the six studies included in this meta-analysis.

First author	Year	Country	Number	Method	HOTAIR expression	Cut-off	Tumour diameter (cm)		Lymphatic node metastasis		Survival
							High versus low		High versus low		
							≤4	>4	Negative Positive		
Jing Li^17^	2015	China	118	RT-qPCR	(7.74, 19.44)	—	—	—	—	—	OS
Hee Jung Kim^15^	2015	Korea	111	RT-qPCR	(22.55, 37.59)	30-fold compared with normal tissues	52 (14)	36 (8)	19 (57)	32 (3)	OS
Zhang Yanmei^14^	2015	China	36	RT-qPCR	(6.625, 7.497)	Median expression of HOTAIR	8 (13)	12 (3)	12 (15)	8 (1)	—
Long Huang^13^	2014	China	218	RT-qPCR	(2.231, 2.535)	Median expression of HOTAIR	70 (88)	39 (21)	68 (103)	41 (6)	OS
Zhang Daosheng^10^	2015	China	32	RT-qPCR	(1.00, 3.56)	—	—	—	—	—	—
Yang Hui^18^	2014	China	20	RT-qPCR	(8.53, 23.27)	—	—	—	—	—	—
